# Fear of Severe Pain Mediates Sex Differences in Pain Sensitivity Responses to Thermal Stimuli

**DOI:** 10.1155/2014/897953

**Published:** 2014-01-05

**Authors:** Maggie E. Horn, Meryl J. Alappattu, Charles W. Gay, Mark Bishop

**Affiliations:** Department of Physical Therapy, University of Florida, P.O. Box 100154, Gainesville, FL 32610, USA

## Abstract

The purpose of this paper was to examine the relationship of sex and pain-related fear in pain intensity reports to thermal stimuli and whether sex differences in reported pain intensity were mediated by pain-related fear. 177 participants, 124 female (23.5 ± 4.5 years old), filled out a demographic and fear of pain questionnaire (FPQ-III). Experimental pain testing was performed using thermal stimuli applied to the lower extremity. Participants rated the intensity of pain using the numerical pain rating scale (NPRS). Independent *t*-tests, Sobel's test, and linear regression models were performed to examine the relationships between sex, fear of pain, and pain sensitivity. We found significant sex differences for thermal pain threshold temperatures (*t* = 2.04,  *P* = 0.04) and suprathreshold pain ratings for 49°C (*t* = −2.12,  *P* = 0.04) and 51°C (*t* = −2.36,  *P* = 0.02). FPQ-severe score mediated the effect of suprathreshold pain ratings of 49° (*t* = 2.00,  *P* = 0.05), 51° (*t* = 2.07,  *P* = 0.04), and pain threshold temperatures (*t* = −2.12,  *P* = 0.03). There are differences in the pain sensitivity between sexes, but this difference may be mediated by baseline psychosocial factors such as fear of pain.

## 1. Introduction

Pain is a prevalent, debilitating condition that has serious health and economic consequences. Approximately 116 million Americans suffer from chronic pain conditions and the costs of pain range from $560 billion to $635 billion annually; this amount is equal to approximately 2,000 dollars for everyone living in the United States [1]. The prevalence of pain in primary care settings is estimated at approximately 30%, with nearly two-thirds of those pain reports attributed to musculoskeletal pain [2].

Evidence suggests men and women experience and report pain differently [3–6]. Clinically this is relevant because research has demonstrated that a greater percentage of chronic pain sufferers are women [4, 7]. Women also report more areas of bodily pain [8, 9] and more pain-related disability compared to men [10]. Given that pain is such a prevalent and debilitating condition with serious health and economic consequences, the Institute of Medicine has stressed the need to improve healthcare delivery of pain management, including individualized treatment approaches [1]. Although an individualized approach to the treatment of pain is recommended, should healthcare providers tailor their treatments based on the sex of the patient or are there other factors that could be important as well?

The etiology of sex differences in pain reports is still not clear. Evidence suggests that different biological [11–14] and psychosocial factors [15–17] may account for these differences. Psychosocial factors influence the perception and evaluation of pain. One commonly studied psychosocial factor is pain-related fear. Pain-related fear includes fear of the sensation of pain, fear of movement or reinjury, and fear of physical activities which are assumed to cause pain [18]. Pain-related fear is believed to contribute to the shift from acute low back pain (LBP) to chronic LBP [19] and numerous studies have demonstrated the association of pain-related fear with disability in patients with chronic [18, 20, 21] and acute [22] LBP, hip and knee osteoarthritis [23, 24], and foot and ankle dysfunction [25]. Camacho-Soto et al. examined the relationship between fear avoidance and disability in older adults and found that higher fear avoidance beliefs were associated with slower gait speeds and higher disability [26]. The results of these studies provide important information about how pain-related fear affects pain perception and functional mobility.

Thus differences in psychosocial factors, such as pain-related fear, across sexes may contribute to differences in the pain reports between men and women. Several lines of research using experimentally induced pain have begun to disentangle these sex differences. For example, an experimental pain study using electrical stimuli found the increased pain experienced by women during a movement task was accounted for by higher reports of fear among women compared to men [27]. In addition to pain-related fear, Robinson et al. [28] have found women to be more willing to report pain and consider themselves to be more sensitive to pain compared to males. Conversely, some males believe that they have higher pain endurance than women and compared to the typical male. Interestingly, after controlling for these gender role differences and anxiety, the previous sex differences in temporal summation, a proxy measure of central sensitization, were attenuated [29]. These findings indicate that sex differences in reported pain may be attributed to underlying differences in psychosocial factors.

This study aimed to investigate the role of pain-related fear and sex on experimentally induced pain using threshold and suprathreshold thermal stimuli. To our knowledge, the influence of pain-related fear as a mediator of sex differences in reported pain intensity to standardized thermal temperatures has not been previously reported. Therefore, the purpose of this paper was to examine sex differences in pain intensity reports to threshold and suprathreshold thermal stimuli, the influence of pain-related fear on pain intensity responses, and whether sex differences in pain intensity reports were mediated by pain-related fear.

## 2. Materials and Methods

### 2.1. Participants

A convenience sample of pain-free participants was pooled from three previously reported studies that tested pain sensitivity and measured pain-related fear [30–32]. Pooling these studies was appropriate for addressing our purposes because the studies had the same eligibility criteria and testing procedures and were all conducted with similar samples (participants were pain-free at the time of testing). All data were collected in the same laboratory and used the same quantitative sensory testing (QST) procedures. Each study had Institutional Review Board approval and all participants provided informed consent before being included in the studies.

Participants provided demographic information and completed a validated psychosocial questionnaire. Participants underwent standardized QST for thermal pain sensitivity at the lower extremity. The QST protocol has been employed in previous studies [30, 31] and is further described below.

### 2.2. Questionnaire

The fear of pain questionnaire (FPQ-III) uses a 30-item, 5-point rating scale to measure fear about specific situations that would normally produce pain [33]. The FPQ-III is a commonly used and well-validated instrument that is appropriate for use in nonclinical and clinical populations [33–35]. The FPQ-III measures fears about pain as a trait-like phenomenon, assessing enduring behavioral patterns across pain situations [33]. The FPQ-III is a multifactor instrument; it can be used to assess fear in a specific area or to evaluate generalization of fear across domains: fear of severe pain, fear of medical pain, and fear of minor pain [33]. Scoring the FPQ-III subscales involves summing the 10 items that comprise each subscale. Items for each subscale are as follows: severe pain = 1, 3, 5, 6, 9, 10, 13, 18, 25, and 27; minor pain = 2, 4, 7, 12, 19, 22, 23, 24, 28, and 30; and medical pain = 8, 11, 14, 15, 16, 17, 20, 21, 26, and 29. The possible range is 10–50 for each subscale. The total score is the sum of all 30 items. The possible range is 30–150 for the total score [33].

### 2.3. Pain Intensity

Intensity of thermally evoked pain was rated using a numerical pain rating scale (NPRS) anchored at one end with 0 “no pain” and at the other end with 100 being “worst imaginable.” Subjects verbally rated their pain after each thermal pulse.

### 2.4. Quantitative Sensory Testing (QST)

All thermal stimuli were delivered to the skin of subjects using a computer-controlled Medoc Neurosensory Analyzer (TSA-2001, Ramat Yishai, Israel).

Before the testing session, each subject underwent a practice session. During this practice session subjects experienced the temperatures to which they were to be exposed. Subjects practiced using the NPRS to rate the intensity of the pain experienced in response to each stimulus. In order to standardize the scaling instructions and to clarify the distinction between the sensory intensity and affective dimensions, a standardized instructional set was used for all subjects during every exposure to the thermal stimuli. The scale instructions were repeated for every set of ratings within each session [36].

#### 2.4.1. Heat Thresholds

After the practice session, heat threshold was measured. The temperature began from a baseline of 32°C. The probe temperature increased at a rate of 0.5°C/s until participants responded that the stimulus was painful. Subjects were then asked to rate that painful sensation using the NPRS. The heat stimuli were applied to the posterior surface of the upper calf below the popliteal fossa, with the subject sitting.

#### 2.4.2. Suprathreshold Pain Ratings

Subjects experienced a sequence of four thermal pulses that included 45, 47, 49, and 51°C presented randomly. Subjects were cued to provide a verbal pain rating of any pain experienced immediately after the peak of each thermal pulse. This procedure was performed twice. The interval between trials was at least 60 seconds to avoid carryover effects from one stimulus to another, to prevent changes in receptor responses and to prevent tissue changes. Temperature levels were monitored by a contactor-contained thermistor and returned to a preset baseline of 35°C by active cooling at a rate of 10°C/sec [37, 38].

### 2.5. Data Analysis

All statistics were analyzed using IBM SPSS Statistics Data Editor 20. Descriptive statistics were calculated for demographic variables. Independent samples *t*-tests were performed to examine the sex differences in FPQ-III scores, threshold temperature, and pain ratings for threshold and suprathreshold pain thermal stimuli. Correlation analyses were performed for FPQ-III questionnaire and pain intensity at threshold and suprathreshold temperatures.

**Figure 1:**
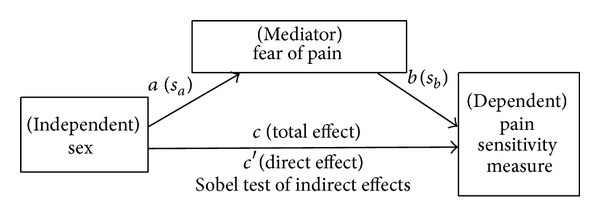
Presumptive mediation model. An illustration of proposed mediation model. *a*, *b*, and *c* are path coefficients. Values in parentheses are standard errors of those path coefficients. Aroian version of the “Sobel test” was used to test indirect effects (equation = *z*-value = *a*∗*b*/SQRT (*b*2∗*sa*2 + *a*2∗*sb*2 + *sa*2∗*sb*2). Key: *a* = raw (unstandardized) regression coefficient for the association between IV and mediator, *s*_*a*_ = standard error of *a*, *b* = raw coefficient for the association between the mediator and the DV (when the IV is also a predictor of the DV); *s*_*b*_ = standard error of *b*.

To address whether pain-related fear accounted for sex differences in pain sensitivity measures, we employed classic mediation analyses in accordance with the methods described by Baron and Kenny (1986) [39] and refined by Preacher and Hayes (2004) [40] using the PROCESS macro for SPSS provided by Hayes [41]. Significance of all indirect effects was assessed with Sobel's statistical test. In our mediation model, sex was the independent variable, pain sensitivity measures were the dependent variable, and fear of pain was the mediator (Figure 1). Prerequisite criteria were established prior to testing for mediation. The three criteria were (1) the mediator needed to be correlated with the independent variable, (2) the outcome variable needed to be correlated with the independent variable, and (3) the mediator needed to be correlated with the outcome.

Separate linear regression analyses were performed to examine the role of sex and fear of pain on experimental pain. Suprathreshold pain ratings at 49°C and 51°C, threshold temperature, and pain ratings at threshold temperature were dependent variables in separate models. Sex, FPQ-Severe, and age were entered as predictors in each of the models.

## 3. Results

**Figure 2:**
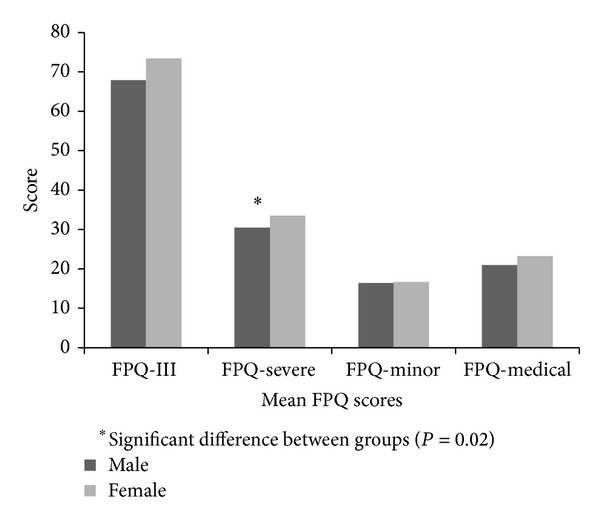
Fear of pain questionnaire domains by sex.

177 participants were included in this analysis. 124 of the participants were female and the mean age of the participants was 23.5 ± 4.5 years old. Participant's FPQ scores are presented as a total score and the subcategories of FPQ-Severe pain, FPQ-minor pain, and FPQ-medical pain. There were no significant differences between sexes for total FPQ-III score (*t* = −1.71, *P* = 0.08), FPQ-minor pain score (*t* = −0.18, *P* = 0.85), or FPQ-medical pain score (*t* = −1.87, *P* = 0.06), but there was a significant difference between sexes on FPQ-severe pain scores (*t* = −2.29, *P* = 0.02), with females reporting higher fear of severe pain than males (see Figure 2).

**Figure 3:**
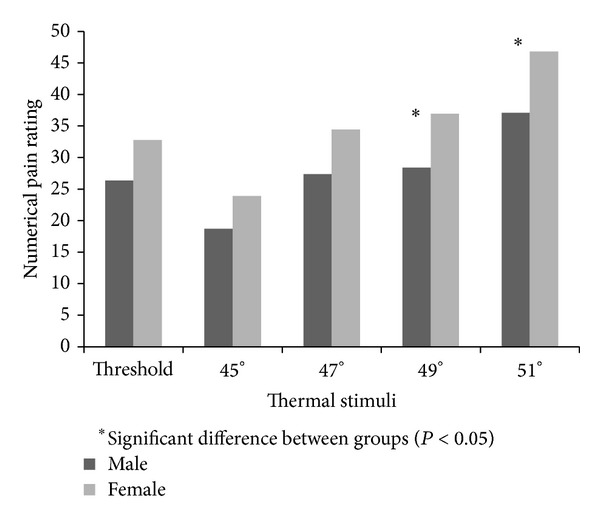
Numerical pain rating in response to thermal stimuli by sex.

We found significant sex differences for thermal pain thresholds temperatures (*t* = 2.04, *P* = 0.04) and suprathreshold pain ratings for 49°C (*t* = −2.12, *P* = 0.04) and 51°C (*t* = −2.36, *P* = 0.02). Females demonstrated lower threshold to thermal stimuli and reported higher pain ratings than males, indicating greater pain sensitivity. However we did not find significant sex differences for pain intensity ratings at 45°C (*t* = −1.43, *P* = 0.15), 47°C (*t* = −1.74, *P* = 0.08), or rating of threshold pain (*t* = −1.90, *P* = 0.06) (see Figure 3).

Correlation analyses revealed that female sex was highly correlated with pain ratings at 49°C (*r* = 0.16, *P* ≤ 0.05) and 51°C (*r* = 0.18, *P* ≤ 0.05) and FPQ-severe score (*r* = 0.17, *P* ≤ 0.05). These correlations indicate higher ratings of thermal stimuli experienced at both 49°C and 51°C and reported fear of pain is correlated with sex. When the correlations of reported fear of pain and pain ratings to thermal stimuli were examined, total FPQ-score and FPQ-severe were correlated with all thermal stimuli pain ratings, with magnitude of the correlation increasing with increasing temperature ratings. FPQ-minor and FPQ-medical were only correlated with suprathreshold ratings at thermal stimuli 49°C and 51°C (see Table 1).

**Figure 4:**
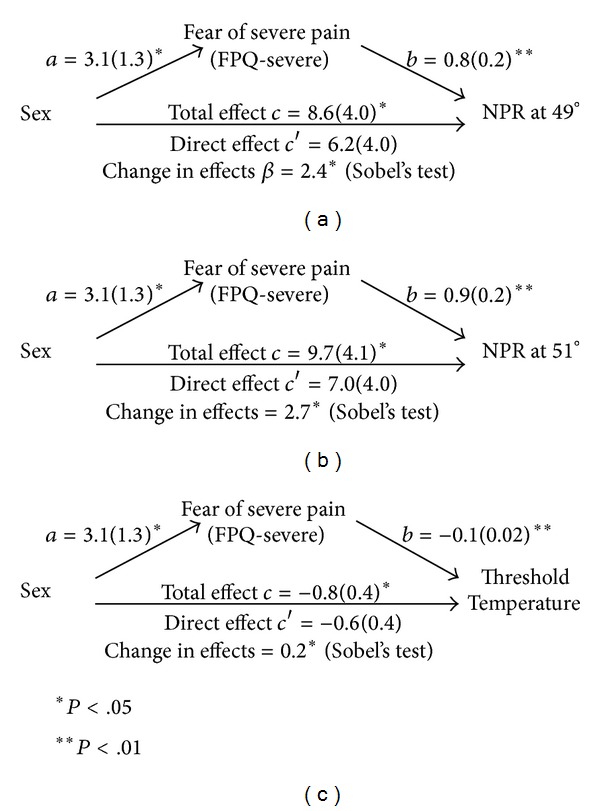
(a)–(c) Results of mediation analyses.

Mediation analyses were carried out for the three pain sensitivity measures demonstrating significant sex differences, which were suprathreshold pain intensity ratings of 49° and 51° and thermal pain threshold temperatures. Prerequisite criteria for mediation were established for fear of severe pain (FPQ-severe). We found that FPQ-fear of severe pain mediated the effect of sex for all three pain sensitivity measures; suprathreshold ratings of 49° (Sobel's *t* = 2.00, SE = 1.24,  and  *P* = 0.05), suprathreshold ratings of 51° (Sobel's *t* = 2.07, SE = 1.35,  and  *P* = 0.04), and thermal pain threshold temperatures (Sobel's *t* = −2.12, SE = 0.15,  and  *P* = 0.03) (see Figures 4(a)–4(c)).

Separate linear regression analyses were performed to explain the variance related to thermal pain ratings and thresholds. These analyses revealed that models containing age, sex, and FPQ-severe score accounted for between 4.5 and 12% of the variance in pain ratings at 45, 47, 49, and 51°C. There was a linear relationship between the magnitude of the effect of the models and temperature of thermal stimulus, where magnitude increased with increasing temperature of the thermal stimulus (45°C (*F*_3,  171_ = 2.65, *P* = 0.51), 47°C (*F*_3,  171_ = 5.18, *P* = 0.002), 49°C (*F*_3,  170_ = 6.20, *P* = 0.001), and 51°C (*F*_3,  172_ = 7.673, *P* < 0.001)). The magnitude of the effect of the models for threshold temperate (*F*_3,  172_ = 4.42, *P* = 0.005) and pain rating at threshold temperature (*F*_3,  172_ = 4.99, *P* = 0.002) were similar (see Table 2).

When specifically examining the effect of sex on dependent variables, sex was not a unique predictor for pain ratings at 45, 47, 49°C, or threshold temperature but was for pain ratings at 51°C (*t* = 1.96, *P* = 0.05) and pain rating at threshold temperature (*t* = 2.27, *P* = 0.03). Sex only accounted for a small amount of variance in these models, 3.2% of the variance in pain rating at 51°C and 2.4% of the variance in threshold temperature pain rating.

FPQ-severe score was a unique predictor in pain ratings at 45°C (*t* = 2.35, *P* = 0.02), 47°C (*t* = 3.24, *P* = 0.001), 49°C (*t* = 3.62, *P* < 0.001), 51°C (*t* = 3.96, *P* ≤ 0.001), and threshold temperature (*t* = −2.59, *P* = 0.01) but not pain rating at threshold temperature (*t* = −1.05, *P* = 0.30). FPQ-Severe accounted for between 3 and 7.6% of variance in each of these models. Age was included in models on a theoretical premise and was not found to be a significant predictor in any model except pain rating at threshold (*t* = 3.26, *P* < 0.001).

## 4. Discussion

The purpose of this study was to investigate sex differences in thermal pain sensitivity measures and the extent to which pain-related fear accounted for these differences. This study adds to the current body of literature as we investigated suprathreshold pain ratings at four standard thermal temperatures and supports that sex differences in pain sensitivity may be accounted for by other factors such as fear of pain. We found that females reported higher fear of severe pain than males and that this difference mediated the difference in pain sensitivity where we found sex differences. Past research examining sex and FPQ scores found similar results where females had significantly higher mean scores than males on the FPQ-severe pain scores [35].

In addition to these findings, females reported statistically significant lower heat thresholds with similar pain intensity ratings at the threshold compared to males. Another interesting observation was that there were no significant differences at the two lower temperatures used in our study. This finding could be interpreted as sex differences are magnified as the intensity of perceived pain increases. This finding may be relevant as the only difference in fear of pain we found in this study was in the severe pain subscale of the FPQ-III.

When controlling for FPQ-severe score and age, sex was only significant in predicting pain ratings at the highest temperature (51°C) and threshold pain rating and sex only accounted for a small amount of variance in these models. These findings indicate that although sex plays a role in suprathreshold and threshold pain ratings, the magnitude is rather small after controlling psychosocial factors. Our data suggest that although sex is not a significant predictor in most models, fear of severe pain is a significant predictor. A greater proportion of variance in all models except pain rating at threshold was explained by FPQ-severe score than by sex. Thus, this finding supports our hypothesis that when painful stimuli are more intense (e.g., highest suprathreshold temperatures), fear and fear of severe pain specifically may play a larger role in pain rating than sex.

This paper highlights the role of fear of pain and sex on pain sensitivity. Although a limitation of this paper is that the findings are in a nonclinical population, the principles can be applicable in conjunction with current knowledge to clinical populations of persons with pain conditions. We know that women and men differ in how they process pain in the experimental setting [42, 43] as well as in the clinical setting [44], but there is still a lack of consistent evidence on the contributions of psychosocial variables, including fear of pain, in the pain experience [45–49]. Past research has found that experimental pain responses were related to clinical pain [8] and treatment outcomes [17]. Thus physicians interested in tailoring pain treatments may be better served by assessing intermediate psychosocial factors that influence an individual's pain perception rather than the sex of the patient alone.

## 5. Conclusion

In summary we found that there are differences in the pain sensitivity between sexes, but this difference may be mediated by baseline psychosocial factors such as fear of pain. Specifically, the fear of severe pain may mediate the effect that sex has on pain intensity ratings in response to thermal stimuli.

## Figures and Tables

**Table 1:** Correlation table of pain ratings to thermal responses with sex and FPQ scores.

	45°C	47°C	49°C	51°C	FPQ-score	FPQ-severe	FPQ-minor	FPQ-medical
Sex-female	0.11	0.13	0.16*	0.18*	0.13	0.17*	0.01	0.14
FPQ-score	0.17*	0.19*	0.24**	0.25**	1	0.91**	0.87**	0.90**
FPQ-severe	0.19*	0.25**	0.28**	0.30**	0.91**	1	0.69**	0.72**
FPQ-minor	0.12	0.15	0.18*	0.19*	0.87**	0.69**	1	0.70**
FPQ-medical	0.15	0.11	0.15*	0.17*	0.90**	0.72**	0.70**	1

**Correlation is significant at the 0.01 level (2-tailed).

*Correlation is significant at the 0.05 level (2-tailed).

**Table 2:** Models for predicting pain ratings at suprathreshold temperatures.

	Variables in the model	Beta	*t*-value	*P*-value	*R* ^2^	Total variance explained by the model (%)
Model 1: pain rating at 45°C	Sex	0.09	1.15	0.25	0.010	4.5%
	FPQ-severe	0.18	2.35	0.02	0.030	
	Age	0.06	0.80	0.43	0.003	

Model 2: pain rating at 47°C	Sex	0.011	1.48	0.14	0.018	8.5%
	FPQ-severe	0.24	3.24	0.001	0.052	
	Age	0.13	1.66	0.09	0.015	

Model 3: pain rating at 49°C	Sex	0.13	1.72	0.09	0.026	10%
	FPQ-severe	0.27	3.62	<0.001	0.067	
	Age	0.89	1.19	0.24	0.007	

Model 4: pain rating at 51°C	Sex	0.15	1.96	0.05	0.032	12%
	FPQ-severe	0.29	3.96	<0.001	0.076	
	Age	0.11	1.52	0.13	0.012	

Model 5: threshold pain rating	Sex	0.17	2.27	0.03	0.021	8.1%
	FPQ-severe	0.08	1.05	0.30	0.003	
	Age	0.25	3.26	0.001	0.057	

Model 6: threshold temperature	Sex	−0.10	−1.36	0.17	0.024	7.3%
	FPQ-severe	−0.20	−2.59	0.01	0.041	
	Age	−0.09	1.20	0.22	0.008	
